# Intimate partner violence: will India find effective solutions?

**DOI:** 10.1016/j.lansea.2023.100147

**Published:** 2023-01-17

**Authors:** Rakhi Dandona

**Affiliations:** a Public Health Foundation of India, Gurugram, India; b Institute for Health Metrics and Evaluation, University of Washington, Seattle, USA

India was recently shaken by the news of gruesome killing of Shraddha Walker allegedly by her live-in partner who strangled her, cut her up into 35 pieces, stored them in a refrigerator before dumping the parts in a forest, and went on with his life until he was caught.^1^ As more details emerged, Shraddha was a victim of intimate partner violence (IPV), which was known to her family, friends and also her employer, and she had even reached out to the police who persuaded her to go home even though she was terrified.^1^^,^^2^ She was also admitted and treated for internal injuries in a hospital but the connect to domestic abuse was not made.^2^ Her friends say that she had feared for her life but still could not walk away from this abusive relationship.

This case highlights monumental failure of the systems and of the society to protect her. The public discourse in India following this killing was centred around three issues – communal and political overtones, victim blaming for her decision to be in a live-in relationship, and the call for justice.^3^^,^^4^ Sadly, bringing inter-faith relationship or victim's choices into the conversation is a tactic to move away from the real issues facing women in India which are the absence of safety net for many women, and failure of the police and health systems to protect them. A recent analysis of nearly 20 years of government data on domestic violence has shown 53% increase in cases filed under ‘cruelty by husband or his relatives’ and a simultaneous decrease in the mean number of persons arrested for these cases between 2001 and 2018.^5^ Moreover, less than 7% of the filed cases had completed legal trial in 2018, and the majority of accused were acquitted in these cases.^5^ These data clearly highlight that protesting and shouting to hang the offender in Shraddha's case is not the solution.^6^ The solution lies in effective implementation of policies and safety net to protect women so that they do not meet such an end. Social safety net for women starts with her family and extends to friends. As evident from Shraddha's case, we need to better understand how these safety nets can actually work in real life to help abused women walk out of abusive relationships, and to seek appropriate care and action. Simply stating that social safety net is needed is clearly not enough if there are no means for it to be translated in real life to save a life.

Though Government of India's commitment towards undertaking reforms to ensure women's rights and elimination of violence against women and girls (VAWG) is reflected in its several legislations and policy reforms, the low budgetary priority weakens the response to VAWG and results in poor quality of services that do not best serve the interests of women.^7^ Shraddha's case suggests that the exposure to IPV can be dynamic and cumulative, and a survivor's response and mental health status will depend on their personal history and life stage, thereby, highlighting the need for a life-course approach to understand IPV to make recommendations for appropriate interventions.^8^^,^^9^ As researchers, clinicians, policy makers and program planners, we will need to take a broad, whole-society view of IPV in India and what should be done to improve the lives of survivors, and how the community can be better prepared to support them. We need more in-depth understanding of where the violence-supportive norms are located within our society, and how and whether the institutional and public structures condone or reinforce these norms.^8^ This would also mean that a significant real effort is needed to effectively address the gender inequality in India's patriarchal society. In addition, we need to invest in more robust measurements of IPV which should involve people with lived experience of IPV and of mental health problems to ensure that the measures generated are relevant, feasible, and valid to guide appropriate and effective interventions.^8^

To conclude, these deaths are inexcusable and preventable. The numbers will simply not go away—we need to act. As India dealt with this horrific crime, news of more gruesome murders poured in.^10^ India needs to wake up now. No women can any longer be left behind.

## Contributors

RD conceptualised and wrote the manuscript.

## Declaration of interests

The author declared no conflicts of interest.
